# Molecular Characterization and the Function of *Argonaute3* in RNAi Pathway of *Plutella xylostella*

**DOI:** 10.3390/ijms19041249

**Published:** 2018-04-20

**Authors:** Muhammad Salman Hameed, Zhengbing Wang, Liette Vasseur, Guang Yang

**Affiliations:** 1 State Key Laboratory of Ecological Pest Control for Fujian and Taiwan Crops, College of Life Science, Fujian Agriculture and Forestry University, Fuzhou 350002, China; msuleman29@gmail.com or msuleman2941@yahoo.com (M.S.H.); lvasseur@brocku.ca (L.V.); 2 Joint International Research Laboratory of Ecological Pest Control, Ministry of Education, Fuzhou 350002, China; 3 Key Laboratory of Integrated Pest Management for Fujian-Taiwan Crops, Ministry of Agriculture, Fuzhou 350002, China; 4 Key Laboratory of Green Pest Control (Fujian Agriculture and Forestry University), Fujian Province University, Fuzhou 350002, China; 5 Key Laboratory of Natural Pesticides and Chemical Biology of the Ministry of Education, South China Agricultural University, Guangzhou 510642, China; wangzi191@yahoo.com; 6 Department of Biological Sciences, Brock University, 500 Glenridge Avenue, St. Catharines, ON L2S 3A1, Canada

**Keywords:** lepidopteran pest, *PxAgo3*, phylogenetic tree, dsRNA, RNAi process

## Abstract

Argonaute (Ago) protein family plays a key role in the RNA interference (RNAi) process in different insects including Lepidopteran. However, the role of Ago proteins in the RNAi pathway of *Plutella xylostella* is still unknown. We cloned an Argonaute3 gene in *P. xylostella* (*PxAgo3*) with the complete coding sequence of 2832 bp. The encoded protein had 935 amino acids with an expected molecular weight of 108.9 kDa and an isoelectric point of 9.29. It contained a PAZ (PIWI/Argonaute/Zwile) domain and PIWI (P-element-induced whimpy testes) domain. PxAgo3 was classified into the Piwi subfamily of Ago proteins with a high similarity of 93.0% with *Bombyx mori* Ago3 (BmAgo3). The suppression of *PxAgo3* by dsPxAgo3 was observed 3 h after treatment and was maintained until 24 h. Knockdown of *PxAgo3* decreased the suppression level of *PxActin* by dsPxActin in *P. xylostella* cells, while overexpression of *PxAgo3* increased the RNAi efficiency. Our results suggest that *PxAgo3* play a key role in the double stranded RNA (dsRNA)-regulated RNAi pathway in *P. xylostella*.

## 1. Introduction

RNA interference (RNAi), also called double stranded RNA (dsRNA)-induced post-transcriptional gene silencing, is a cellular mechanism regulated by a unique group of RNA-binding proteins named Argonautes (Ago) [1,2]. Ago proteins bind to small administrative RNAs, such as small interfering RNA (siRNA), micro RNA (microRNA), and Piwi-interacting RNA (piRNA), to generate the RNA-induced silencing complex (RISC) [3]. Each Ago protein consists of two fundamental domains, PAZ (PIWI/Argonaute/Zwile) and PIWI (P-element-induced wimpy testes), in their structure [4]. Ago protein structures of eukaryotes and prokaryotes share a bilobed architectural structure containing four domains of N, PAZ, MID (middle) and, PIWI connected by two linkers [5]. The N domain is linked with a small RNA duplex loosening up during the RISC formation [6]. The PAZ domain contains an oligonucleotide-binding fold at the 3′-end of RNA and can bind to siRNAs [7]. The MID domain generates an associating pocket for fixing 5′-phosphate of the small RNAs [8], while PIWI domain is orthologous to ribonuclease H (RNase H) and encloses the slicing residues Asp-Asp-His (DDH), which is significant for the endonucleolytic “cleavage” action of the RISC [9].

The number of Ago proteins varies among different insect species. For example, two Ago proteins have been reported in *Anopheles gambiae* [10], three in *Leptinotarsa decemlineata* [11], four in *Bombyx mori* [12], and five in *Drosophila melanogaster* [13]. Ago proteins are divided into three groups of Piwi subfamily, Ago subfamily, and worm-specific Argonautes (WAGO) [14]. In *Penaeus monodon*, PmAgo3 is found within the Ago subfamily [9], while in *D. melanogaster* [15] and *B. mori* [16], the Ago3 protein is placed in the Piwi subfamily. Ago3 protein is related to the piRNA-mediated pathway and silences transposable elements (TEs) in *D. melanogaster* [17]. The sense piRNAs bind to Ago3 to cleave the primary piRNA precursors [18]. Ago3 protein is located in the cytoplasm of *D. melanogaster* germ cells, where it works in the Ping-Pong cycle to destroy transposon mRNAs [19]. In humans, Ago3 cleaves in a similar pattern as Ago2 protein [20], and up-regulates the ratio between passenger strand and guide strand of miRNA let-7a [21]. In *Tribolium castaneum*, Ago3 is homologous with DmAgo3 and is involved in transcriptional silencing mechanism [22]. In *D. melanogaster* ovary, transposon elements are suppressed by Ago3 protein [23]. DmAgo3 knockdown in embryos shows an increase in both telomeric transposition and telomere length [24]. Ago3 is also recognized as a slicer protein and, in insects, it is involved in piRNA-mediated gene silencing [25]. The overexpression of Ago3 increases the RNAi efficiency in Lepidopteran cell lines [26], *Caenorhabditis elegans* [14], and *H. sapiens* [21]. 

*Plutella xylostella* (diamondback moth) is a worldwide pest of cruciferous plants that has become a model species due to its high reproductive rate, fast dispersal, and capacity to develop resistance to most classes of pesticides [27,28]. Previous studies have shown the phenomenon of RNAi in *P. xylostella* larvae [29]. Here, we identified and described the *Ago3* gene in *P. xylostella* (*PxAgo3*) and its function in the RNAi pathway of *P. xylostella*. The expression of *PxAgo3* in response to dsPxAgo3 challenges were investigated in *P. xylostella* larvae. We then evaluated the effects of knockdown or overexpression of *PxAgo3* on the suppression of targeting gene, *PxActin* by dsPxActin in *P. xylostella* cells. Our results suggest that *PxAgo3* plays a significant role in the RNAi pathway of *P. xylostella.*

## 2. Results

### 2.1. Identification and Sequence Analysis of PxAgo3 Gene

**Figure 1:**
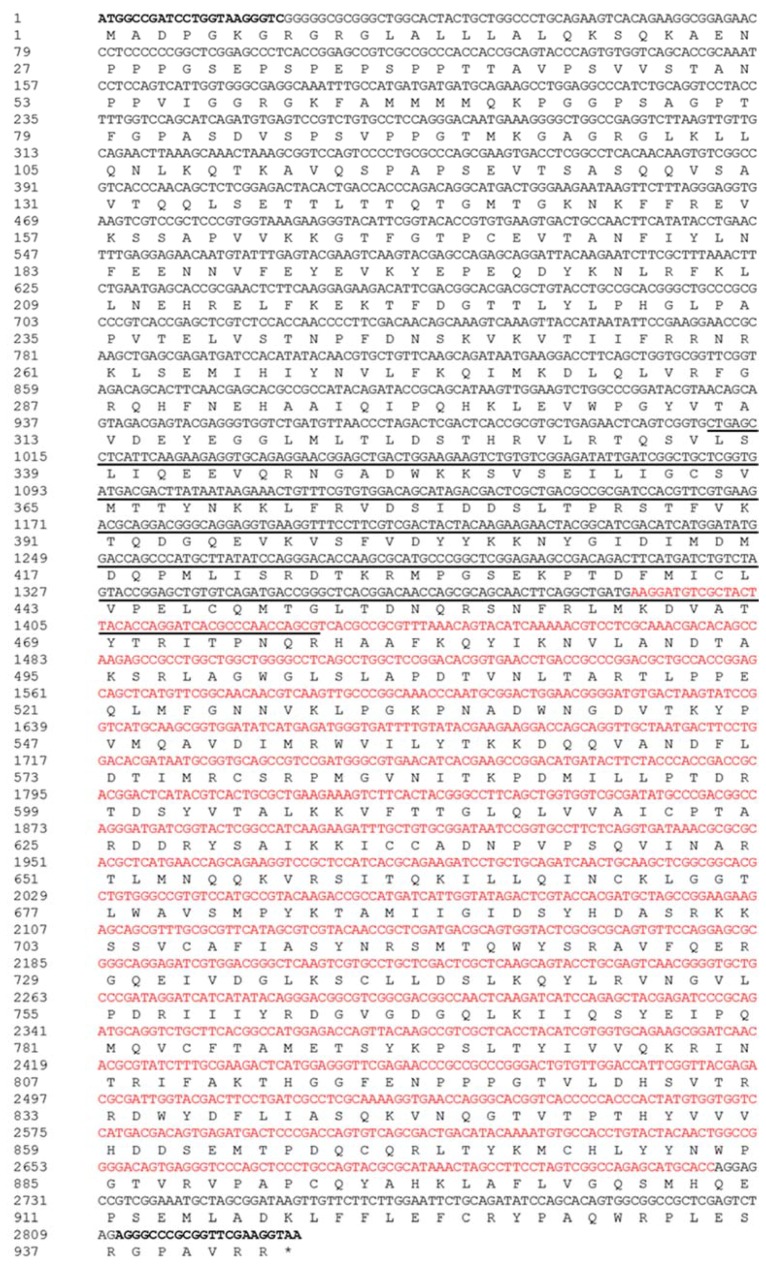
The complete coding sequence and the assumed amino acid sequence of *PxAgo3*. The coding sequence is of 2832 bp, starting from ATG marked in boldface and ending with TAA denoted with asterisk (*). The protein sequence is of 943 aa under the nucleotide sequence, with two motifs of the PAZ domain (underline, position 337–477) and the PIWI domain (red in color, position 464–909). Besides this, PCR primers (sense and antisense) are highlighted in bold.

Using Ago3 CDS sequence from *B. mori* (BmAgo3, GenBank: AB372007.1), we identified Px011435 as the Ago3 coding sequence in *P. xylostella* genome. Using the specific primers of PxAgo3-F and PxAgo3-R (Table 1) based on Px011435, *PxAgo3* was amplified through PCR and sequenced. The sequence was deposited in the GenBank with the accession number of MG778697. The sequence of *PxAgo3* was of 2832 bp with the predicted protein of 943 aa (Figure 1B). The cloned sequence contained two conserved regions with the PAZ domain of 420 bp in length and the Piwi domain of 1335 bp from 3′ end to 5′ end. 

**Figure 2:**
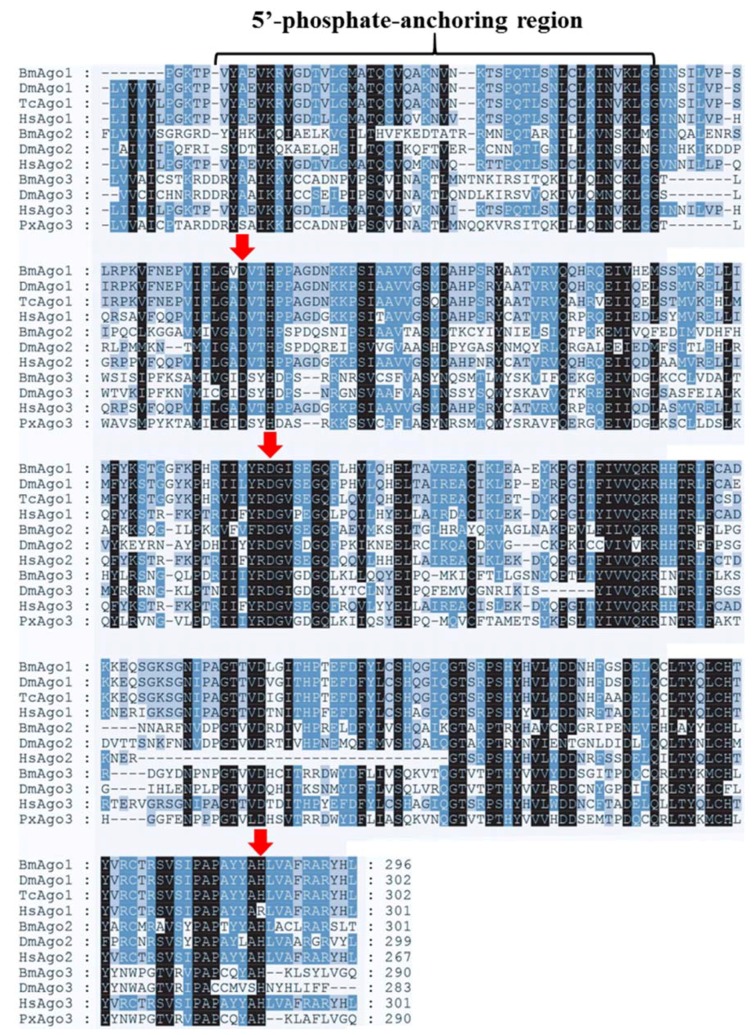
Multiple-sequence alignment of the PIWI domain of *PxAgo3* with those of other species. Multiple-sequence alignment of the PIWI domains of *PxAgo3* and descriptive Ago proteins by using the ClustalW software (Omega, Bienne, Switzerland). The undistinguishable amino acids are marked in black shadow, whereas alike amino acids are denoted in blue and grey shadow. The location of catalytic residues of DDH motif are specified with red arrows. Bm means *Bombyx mori*; Dm, *Drosophila melanogaster*; Tc, *Tribolium castaneum*; Hs, *Homo sapiens*; Px, *Plutella xylostella*.

**Figure 3:**
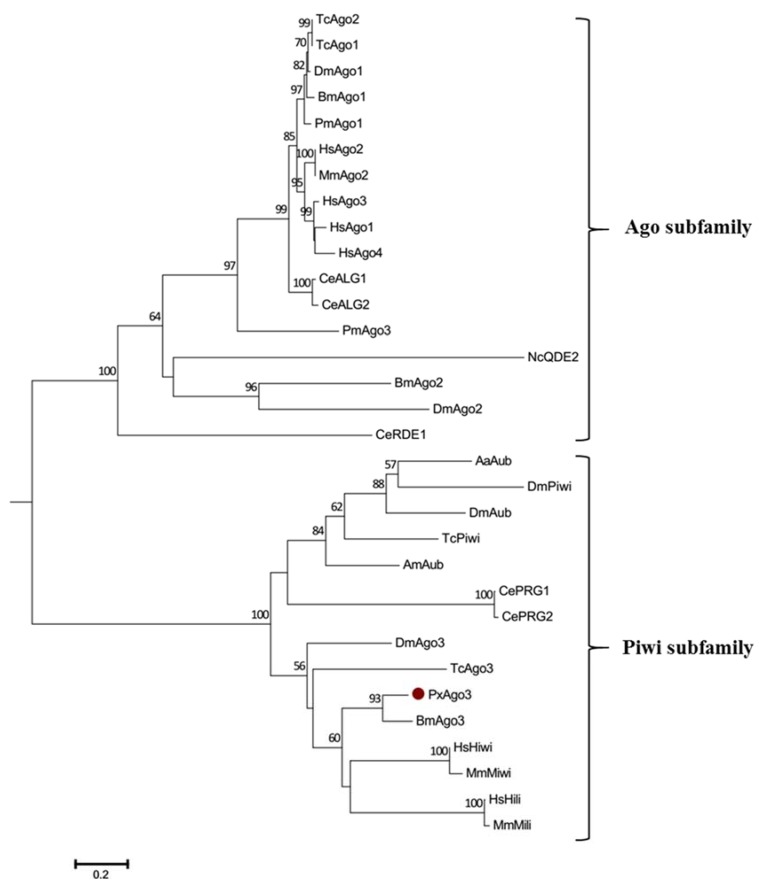
Phylogenetic tree of Ago proteins. The neighbor-joining tree was developed by using the MEGA 6.0 software (Molecular Evolutionary Genetics Analysis, Tokyo, Japan). The abbreviations are listed in Table 2. The bootstrap values of 1000 repeats are shown at the nodes.

GenBank database sequence comparison showed that PxAgo3 was similar to *B. mori* Piwi-like Ago3 protein with the similarity of 49.3%. To analyze the functional sequence, the deduced amino acid sequence of PxAgo3 and some expressive Ago family protein sequences were aligned based on the conserved PIWI domain. The multiple-sequence alignment led to the identification of one 5′-phosphate-anchoring region and three catalytic residues (DDH positioned by red arrows, Figure 2) inside the PIWI domain. Thirty-one Ago proteins sequences from ten different species were collected through the NCBI website to construct the phylogenetic tree (Table 2). The phylogenetic tree showed that the Ago proteins were divided into two subfamilies, Ago- and Piwi, with PxAgo3 in the Piwi subfamily located with BmAgo3 (Figure 3).

### 2.2. PxAgo3 Expression in Response to dsRNA in P. xylostella Larvae

**Figure 4:**
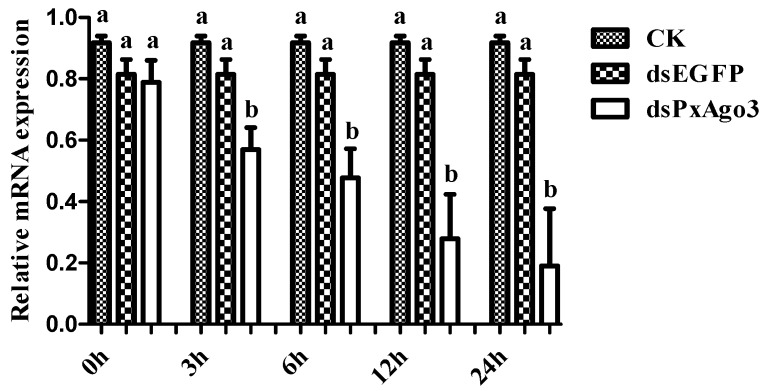
Comparison of *PxAgo3* suppression levels in *P. xylostella* larvae by dsPxAgo3 at different times after treatment. Different lowercase letters represent significant differences among treatments at each time interval (Tukey’s post hoc test, *p* < 0.05).

The expression of *PxAgo3* was significantly more suppressed by dsPxAgo3 in the 3rd instar larvae of *P. xylostella* than by CK and dsEGFP. The suppression was first observed in 3 h after treatment and maintained up to 24 h (Figure 4).

### 2.3. PxAgo3 Suppression Decreased the RNAi Efficiency in P. xylostella Cells

**Figure 5:**
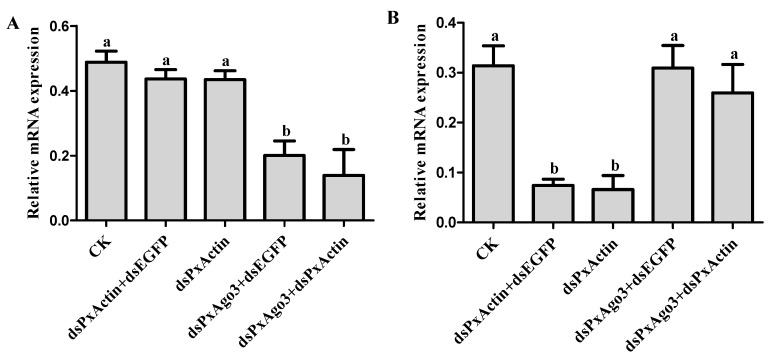
Effects of PxAgo3 knockdown on *PxActin* suppression in *P. xylostella* cells. (**A**) *PxAgo3* expression; (**B**) *PxActin* expression. Different lowercase letters represent significant differences among treatments at each time interval (Tukey’s post hoc test, *p* < 0.05).

We hypothesized that the knockdown of *PxAgo3* by dsPxAgo3 would affect the suppression of endogenous gene *PxActin* by dsPxActin. Silencing of *PxAgo3* by dsPxAgo3 led to a significant down-regulation (*F* = 11.34, *df*_1_ = 4, *df*_2_ = 10, *p* = 0.0010) (Figure 5A). For RNAi of RNAi, *P. xylostella* cells were exposed to dsPxAgo3 for 24 h, followed by exposure of these cells to dsPxActin for another 24 h. *PxActin* expression was significantly suppressed by dsPxActin and dsPxActin+dsEGFP, but not by dsPxAgo3+dsPxActin (*F* = 9.91, *df*_1_ = 4, *df*_2_ = 10, *p* = 0.0017) (Figure 5B). These results demonstrated that the dsRNA-mediated suppression of *PxAgo3* reduced the RNAi efficiency in *P. xylostella* cells.

### 2.4. PxAgo3 Overexpression Increased the RNAi Efficiency in P. xylostella Cells

**Figure 6:**
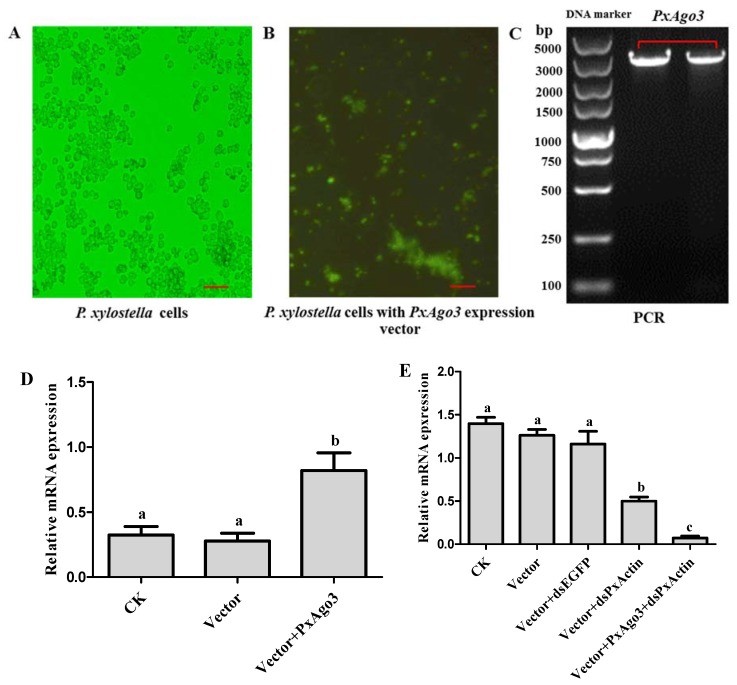
Effects of *PxAgo3* overexpression on *PxActin* suppression in *P. xylostella* cells. (**A**) *P. xylostella* cells; (**B**) the phenotypical expression of *PxAgo3* expression vector into *P. xylostella* cells. Scale Bar: 100 µm; (**C**) PCR product of *PxAgo3* using *P. xylostella* cells with *PxAgo3* expression vector as the template; (**D**) the expression level of *PxAgo3* in *P. xylostella* cells; (**E**) the expression level of *PxActin* in *P. xylostella* cells. Different lowercase letters represent significant differences among treatments (Tukey’s post hoc test, *p* < 0.05)

To overexpress *PxAgo3* in *P. xylostella* cells, the expression construct was inserted into the PIZT/V5 vector and transfected into *P. xylostella* cells. The success of transfection was verified by the enhanced green fluorescence protein (EGFP) expression with the green fluorescence observed under the florescence microscope (Figure 6A,B) and by PCR using vector-based primers (Figure 6C). The expression level of *PxAgo3* in *P. xylostella* cells significantly increased in transfected *P. xylostella* cells (*F* = 10.45, *df*_1_ = 2, *df*_2_ = 6, *p* = 0.0111) (Figure 6D). To observe the RNAi efficiency in *PxAgo3*-overexpressed cells, *PxActin* was targeted to suppress in *PxAgo3*-overexpressed cells. The *PxActin* expression was affected by the treatments (*F* = 45.95, *df*_1_ = 4, *df*_2_ = 10, *p* < 0.0001). *PxActin* expression was reduced by dsPxActin in normal cells and the suppression level was increased in *PxAgo3*-overexpressing cells (Figure 6E). These results suggested that overexpression of *PxAgo3* increased the dsRNA-regulated RNAi efficiency in *P. xylostella* cells.

## 3. Discussion

In this study, we have cloned and identified a new Ago gene from *P. xylostella*, *PxAgo3*. The amino acid sequence of *PxAgo3* included a PAZ domain and a PIWI domain. PAZ and PIWI are similar in different species, such as *P. monodon* [9], *B. mori* [16], *D. melanogaster* [15]. The PAZ domain is a particular nucleic acid binding site, which plays a significant role in RNAi mechanism [7]. Ago3 is physically bound with nucleic acid through the PAZ domain, while the PIWI domain interacts with the 5′ end of the guide RNA in *Archaeoglobus fulgidus* [30]. The three catalytic residues (DDH) within the PIWI domain of Ago3 form a cleavage triad in DmAgo3 protein [31], in BmAgo3 protein [32], and in PmAgo3 protein [9], and was also observed in PxAgo3.

The phylogenetic tree showed that PxAgo3 protein belongs to the Piwi subfamily. Ago3 has been reported to belong to the Piwi subfamily in *B. mori* [16,32,33], *D. melanogaster* [22], and *Aedes aegypti* [34]. however, Ago3 belongs to the Ago subfamily in *C. elegans* [14,35] and *H. sapiens* [36,37].

The silencing of *PxAgo3* occurred 3 h after dsRNA treatment and was maintained for 24 h. *Ago3* knockdown is observed 24 h after introduction of dsRNA in mammalian cells [38] as well as *A. aegypti*, and *A. albopictus* cells [39]. In *Culex quinquefasciatus, Ago3* suppression is only observed 48 h after treatment [40]. Therefore, it is suggested that *Ago3* can be effectively suppressed by dsRNA in animals.

PxAgo3 knockdown reduced RNAi efficiency in *P. xylostella* cells. Ago3 is involved in dsRNA-regulated RNAi phenomenon [22] and reduces RNAi response in *D. melanogaster* [41] and *B. mori* [42]. Ago2 has been considered the only cleavage component in siRNA-regulated RNAi process in insects [42,43]. Our results, however, suggest that Ago3 may be involved in the siRNA-regulated RNAi pathway, which is known to be associated with the piRNA pathway. The overexpression of PxAgo3 also enhanced the RNAi response. The overexpression of Ago3 in Lepidopteran cell lines [26], *C. elegans* [14], as well as HEK293 and HeLa cells [21] can increase the RNAi efficiency. It is possible that PxAgo3 overexpression may facilitate the dsRNA-mediated RNAi in *P. xylostella.*

In conclusion, we sequenced *PxAgo3* and identified PxAgo3 as a Piwi subfamily member containing the cleavage motif of DDH in the PIWI domain. We discovered that Ago3 is involved in the siRNA-mediated pathway in *P. xylostella* by decreasing PxActin suppression by dsPxActin through knockdown of PxAgo3 and enhancing PxActin suppression by dsPxActin through overexpression of PxAgo3 in *P. xylostella* cells.

## 4. Materials and Methods

### 4.1. P. xylostella Fuzhou-S Rearing and Cell Culture

The *P. xylostella* individuals used in this study came from an insecticide-susceptible strain previously used for *P. xylostella* genomic sequencing [44]. *P. xylostella* larvae were reared on radish seedlings at 25 ± 1 °C, 65 ± 5% RH and 16 h L: 8 h D. A stable *P. xylostella* cell line was used, which was established from embryonic tissues of *P. xylostella* [45]. Several generations of *P. xylostella* cells were maintained at 27 °C in Grace’s medium (Invitrogen, Waltham, MA, USA) supplemented with 10% of fetal bovine serum (FBS).

### 4.2. Total RNAs Purification and cDNA Preparation

Total RNAs were purified from four 3rd instar larvae of *P. xylostella* using the TRIzol-Reagent (Invitrogen). The quantity of RNAs were measured using a Nanodrop ND-1000 spectrophotometer (Invitrogen), and the RNA quality was verified through 1% gel electrophoresis. Total RNAs were diluted with nuclease-free water (Promega, Madison, WI, USA) and maintained at a concentration of 500 ng/μL. Furthermore, 1 μL of purified RNA was used as the template for cDNA preparation by using the GoTaq^®^ 2-Step RT-qPCR System (Promega).

### 4.3. Molecular Cloning and Expression Vector Construction of PxAgo3

For *PxAgo3* cloning, a specific pair of primers (PxAgo3-F and PxAgo3-R) (Table 1) were designed using the Primer Premier 5 according to the gene sequence of *PxAgo3* (gene ID: Px011435) from *P. xylostella* genome (Available online: http://iae.fafu.edu.cn/DBM/index.php). The Phanta Max Super-Fidelity DNA Polymerase (Vazyme, Nanjing, China) was used to conduct PCR following the manufacture protocol and conditions: denaturation at 95 °C for 3 min, amplification by 30 cycles at 95 °C for 15 s, 56 °C for 15 s, and 72 °C for 3 min, the last expansion at 72 °C for 10 min and then storage at 4 °C. The PCR product was extracted from the gel by using the QIAquick Gel extraction kit (Qiagen, Venlo, The Netherlands) according to the manufacturer protocol. The extracted PCR product and PJET vector were ligated using T4 DNA ligase enzyme (Promega) according to the ClonJET PCR Cloning kit (Invitrogen) and incubated at 25 °C for 1 h. The ligated sample was mixed with 50 μL high efficiency competent cells of DH-5α (Tiangen, Beijing, China) and incubated on ice for 30 min. The sample was cultured in the selection media to obtain white colonies. The colony PCR was done using the Go Taq^®^ Green Master Mix enzyme (Promega) with the vector-based primers (Table 1). The PCR product was verified through 1% gel electrophoresis following the procedure previously described. Eight white colonies were checked and one verified positive white colony was cultured. An amount of 500 µL of the cultured solution was sent to General Biosystems (China) for sequencing. The protein sequence of PxAgo3 was blasted in the National Center for Biotechnology Information (NCBI) (Available online: https://blast.ncbi.nlm.nih.gov/Blast.cgi?PROGRAM=blastp&PAGETYPE=BlastSearch&LINK_LOC=blasthome) to verify its identity and submitted to GenBank (Available online: https://submit.ncbi.nlm.nih.gov/subs/genbank/) for accession number.

To construct the *PxAgo3* expression vector (PIZT/V5-His+PxAgo3), the PCR product and PIZT/V5-His were individually double-digested at 37 °C using two restriction enzymes of FastDigest KpnI and EcoRI (Invitrogen). Both double digested products (PCR product and PIZT/V5-His) were ligated and transfected into competent cells and checked by colony PCR and 1% gel electrophoresis (as previously described). A positive white colony cultured solution of *PxAgo3* expression vector was extracted using the Hispeed Plasmid Midi kit (Qiagen).

### 4.4. Phylogenetic Tree and Sequence Analysis

Ago protein sequences were downloaded from the GenBank database of NCBI (Available online: https://www.ncbi.nlm.nih.gov/) and are listed in Table 2. A multiple-sequence alignment was performed based on the PIWI domain using the ClustalW software. The phylogenetic tree was constructed using MEGA 6.0 based on the neighbor-joining method with a bootstrap value of 1000 replicates. The isoelectric point and molecular weight of PxAgo3 were identified using the Expasy website (Available online: http://www.expasy.org).

### 4.5. Double-Stranded RNA (dsRNA) Preparation

The following gene-specific sequences were used as the dsRNA templates: 389-bp fragment targeting *PxAgo3*, 461-bp fragment targeting *PxActin*, and 457-bp fragment targeting *EGFP*. DsRNAs were prepared using the Ambion MEGAscript RNAi kit (Invitrogen). The primers listed in Table 1 were used to amplify these fragments with the T7-polymerase promoter (TAATACGACTCACTATAGGG) sequence. The reaction mixture was incubated at 37 °C for 3 h, heat shocked at 75 °C for 5 min and then kept at room temperature for 1 h for the annealing reaction. Subsequently, dsRNAs were treated with DNase I to remove the template DNA, purified with the phenol/chloroform method and finally diluted in nuclease-free water. The dsRNA quality and quantity were measured following the procedure previously described.

### 4.6. DsRNA Injection and Transfection into P. xylostella Larvae and Cells

For dsRNA injection, 3rd instar larvae of *P. xylostella* were selected and borosilicate micro-capillaries (Glass Replacement 1.1) were pulled using heater setting 64 in PC-10 (Narishige, Tokyo, Japan). We injected 10 μL dsRNAs (1 μg) of *PxAgo3, PxActin* and EGFP (control) by using micro-injection world precision instruments (Sarasota, FL, USA) into the hemolymph of 3rd instar larvae (*n* = 60) individually. After injection, larvae were transferred into separate plastic rearing box and fed with fresh radish leaves. Purified dsRNAs were transfected into the *P. xylostella* cells using the Cellfectin^®^ II reagent (Promega) according to the manufacturer’s instructions.

### 4.7. RNAi of RNAi Assay in P. xylostella Cells

For RNAi of RNAi assay, one day before transfection of dsRNAs into cells, *P. xylostella* cells were cultured in 2 mL of Sf-900 III serum free media (SFM) (Promega) with 10% fetal bovine serum (FBS) (HyClone, Utah, United States) and containing 1% anti-toxin antimycotic (pH 6.2) at a concentration of 6 × 10^5^ cells/mL, in 6-well plates (Corning Incorporated, New York, NY, USA). Cells were incubated at 27 °C overnight or until 50–60% of the cells converged. We transfected two types of dsRNA into *P. xylostella* cells in order. Firstly, 1 μg of dsPxAgo3 was transfected into *P. xylostella* cells by following the Cellfectin^®^ (Gibco Invitrogen, Waltham, MA, USA) manufacturer’s instructions. The treated *P. xylostella* cells were incubated at 27 °C for 24 h in a closed chamber to prevent environmental contamination. Secondly, 1 μg of dsPxActin was transfected using the Cellfectin reagent (described as before) into the same *P. xylostella* cells 24 h after the first transfection of dsPxAgo3. The transfection of dsEGFP was used as a control. The *PxAgo3* expression level was analyzed by using RT-qPCR. Similarly, the overexpression of *PxAgo3* was measured after transfection of PIZT/V5-His+PxAgo3 into *P. xylostella* cells by RT-qPCR. The presence of vectors was examined through green fluorescence by using a fluorescence microscope Olympus IX51 (Olympus Corporation, Tokyo, Japan).

The cells were collected 48 h post transfection in 1.5 mL tubes (Axygen Incorporation, Tewksbury, MA, USA) and centrifuged at 1500× *g* for 5 min at 4 °C. Total RNA extraction, quality and quantity verifications, and cDNA preparation were performed as previously described. *PxAgo3* and *PxActin* expression levels were monitored through RT-qPCR using the primers listed in Table 1. All procedures were done with three biological replicates.

### 4.8. RT-qPCR Analysis

RT-qPCR was performed using the GoTaq^®^ qPCR master mix (Promega). RT-qPCR was performed according to the following conditions: at 95 °C for 3 min for denaturation, followed by 40 cycles at 95 °C for 5 s and 60 °C for 20 s. The degree of amplification was determined at the end of each cycle by fluorescence intensity measurements. Quantitative mRNA measurements were done in triplicate and standardized to an internal control of ribosomal protein 32 (*RL-32*) (reference gene). All of the RT-qPCR primers are listed in Table 1.

### 4.9. Statistical Analysis

One-way analysis of variance (ANOVA) was done using the GraphPad (version 5.01) (GraphPad Software, La Jolla, San Diego, CA, USA), and then differences between treatments were determined using Tukey test at *p* < 0.05.

## Figures and Tables

**Table 1:** Primers used in the three experiments of vector construction, expression and suppression for *PxAgo3.*

Primer	Sequence	Use
PxAgo3-F	5′ ATGGCCGATCCTGGTAAGGGTC 3′	PCR
PxAgo3-R	5′ TTACCTTCGAACCGCGGGCCCT 3′	PCR
PxAgo3 KpnI-F1	5′ CGGGGTACCATGGCCGATCCTGGTAAGG 3′	PCR
PxAgo3 EcoRI-R1	5′ CCGGAATTCCAAGAAGAACAACTTATCCGC 3′	PCR
PIZT-F	5′ CGCAACGATCTGGTAAACAC 3′	PCR
PIZT-R	5′ CTGACTAAATCTTAGTTTGTATTGTC 3′	PCR
PxAgo3-F1	5′ GCCGCCATACAGATACCG 3′	RT-qPCR
PxAgo3-R1	5′ AACATCAGACCACCCTCG 3′	RT-qPCR
PxActin-F2	5′ GGAGTGATGGTCGGTATGGGA 3′	RT-qPCR
PxActin-R2	5′ CTCGATGGGGTACTTCAGGGT 3′	RT-qPCR
RL32-F	5′ CAATCAGGCCAATTTACCGC 3′	RT-qPCR
RL32-R	5′ CTGGGTTTACGCCAGTTACG 3′	RT-qPCR
dsAgo3-F	5′ GTCCGCTCCATCACGCAGAAA 3′	dsRNA Preparation
dsAgo3-R	5′ GCGGGATCTCGTAGCTCTGGA 3′	dsRNA Preparation
dsActin-F	5′ GCCAACCGTGAAAAGATGA 3′	dsRNA Preparation
dsActin-R	5′ AGGAATGAGGGCTGGAACA 3′	dsRNA Preparation
dsEGFP-F	5′ GTGTTCAATGCTTTTCCCGTTATCC 3′	dsRNA Preparation
dsEGFP-R	5′ ACCATGTGGTCACGCTTTTCG 3′	dsRNA Preparation

Note: PCR, polymerase chain reaction, RT-qPCR, quantitative reverse transcription PCR; dsRNA, double stranded RNA.

**Table 2:** Ago proteins with the accession number for multiple-alignment comparison and phylogenetic tree analyses.

Organism	Protein	Abbreviation	Acc. Number
*Tribolium castaneum*	Argonaute-1	TcAgo1	KYB26000.1
	Argonaute-2	TcAgo2	XP_015837988.1
	Argonaute 3	TcAgo3	EFA02921.1
	PIWI	TcPIWI	EFA07425.1
*Drosophila melanogaster*	Argonaute-1	DmAgo1	AAF58313.1
	Argonaute-2	DmAgo2	NP_730054.1
	Argonaute-3	DmAgo3	EAA45981.3
	Aubergine	DmAub	CAA64320.1
	PIWI	DmPIWI	AGL81541.1
*Bombyx mori*	Argonaute-1	BmAgo1	NP_001095931.1
	Argonaute-2	BmAgo2	BAD91160.2
	Argonaute-3	BmAgo3	A9ZSZ2
*Caenorhabditis elegans*	CeALG-1	CeALG1	CAR97837.1
	CeALG-2	CeALG2	CCD73271.1
	CePRG-1	CePRG1	CAA98113.1
	CePRG-2	CePRG2	CCD62443.1
	CeRDE-1	CeRDE1	CAB05546
*Homo sapiens*	Argonaute-1	HsAgo1	NP_036331.1
	Argonaute-2	HsAgo2	NP_036286.2
	Argonaute-3	HsAgo3	NP_079128.2
	Argonaute-4	HsAgo4	NP_060099.2
	HsHiwi	HsHiwi	AAC97371.2
	HsHili	HsHili	NP060538.2
*Penaeus monodon*	Argonaute-1	PmAgo1	ABG66641.1
	Argonaute-3	PmAgo3	AGC95229.1
*Apis mellifera*	Aubergine	AmAub	NP_001159378.1
*Aedes aegypti*	Aubergine	AaAub	XP_021707306.1
*Neurospora crassa*	NcQDE-2	NcQDE2	AAF43641
*Mus musculus*	Argonaute-2	MmAgo2	NP_694818
	MmMiwi	MmMiwi	BAM37459
	MmMili	MmMili	BAA93706
*Plutella xylostella*	Argonaute-3	PxAgo3	MG778697
